# Cross-cultural adaptation and psychometric evaluation of the artificial intelligence learning intention scale (AILIS) among medical sciences students: a methodological study

**DOI:** 10.1080/10872981.2026.2690326

**Published:** 2026-06-18

**Authors:** Amir Hossein Taghiparast, Amaneh Mahmoudian, Maryam Ghaemi-Amiri, Fatemeh Ghaffari

**Affiliations:** a Student Research Committee, Nursing Care Research Center, Health Research Institute, Babol University of Medical Sciences, Babol, I.R. Iran; b Nursing Care Research Center, Health Research Institute, Babol University of Medical Sciences, Babol, I.R. Iran; c Social Determinants of Health Research Center, Health Research Institute, Education Development Center, Department of Medical Sciences Education Development, Babol University of Medical Sciences, Babol, Iran; d Department of Nursing, Faculty of Medical Sciences, Tarbiat Modares University, Tehran, Iran

**Keywords:** Instrument validation, medical education, technology acceptance, student motivation, psychometric assessment

## Abstract

**Background:**

This study aimed to adapt and psychometrically evaluate the Artificial Intelligence Learning Intention Scale (AILIS) among Iranian medical sciences students.

**Methods:**

This methodological psychometric study was conducted among 800 medical sciences students at Babol University of Medical Sciences, Iran. The AILIS was translated and culturally adapted into Persian using a standardized cross-cultural adaptation process. Construct validity was examined through exploratory factor analysis (EFA) and confirmatory factor analysis (CFA). Reliability was assessed using Cronbach’s alpha, McDonald’s omega, and the intraclass correlation coefficient (ICC). Convergent and discriminant validity were evaluated using composite reliability (CR) and average variance extracted (AVE). Measurement invariance across gender and structural stability were assessed using multi-group confirmatory factor analysis and exploratory graph analysis (EGA).

**Results:**

EFA supported a four-factor structure consisting of Epistemic Capacity, Psychological Attitudes, Facilitating Environments, and Psychological and Behavioral Outcomes, explaining 44.55% of the total variance, with factor loadings greater than 0.40. Confirmatory factor analysis indicated an acceptable model fit, supporting the proposed factor structure (χ²/df = 3.42, RMSEA = 0.078, CFI = 0.932). Internal consistency and test–retest reliability were satisfactory, with Cronbach’s alpha ranging from 0.854 to 0.904, McDonald’s omega from 0.854 to 0.899, and ICC from 0.750 to 0.860. Composite reliability values were adequate, while AVE values indicated acceptable but partially limited evidence of convergent validity. Discriminant validity was generally supported. In addition, multi-group analysis demonstrated measurement invariance across gender, and exploratory graph analysis confirmed the structural stability of the four-factor model.

**Conclusion:**

The Persian AILIS is a valid and reliable instrument for assessing AI learning intention among medical sciences students. Beyond its psychometric robustness, the instrument may support curriculum planning, evaluation of AI-related educational interventions, and evidence-based decision-making in medical sciences education by providing a standardized measure of students’ intentions to engage with AI learning.

## Introduction

Artificial intelligence (AI) has emerged over the past decade as a transformative force in healthcare systems and medical sciences education. Rapid advances in machine learning, natural language processing, computer vision, and big data analytics have enabled the application of intelligent algorithms in disease diagnosis, clinical decision-making, outcome prediction, healthcare resource management, and medical education [1,2]. These developments have not only reshaped the delivery of healthcare services but have also generated new expectations for medical education systems to prepare graduates with technological literacy, digital critical thinking skills, and the ability to effectively engage with intelligent systems [2,3]. Within this evolving educational landscape, developing medical sciences students’ competencies, readiness, and willingness to engage with AI technologies in a critical, ethical, and evidence-informed manner has become an increasingly recognised priority in medical education curricula worldwide [3,4].

In response to these developments, medical and health sciences education programmes worldwide have increasingly integrated AI-related competencies into their curricula through formal coursework, workshops, simulation-based learning, and clinical training experiences [2,3]. However, emerging evidence suggests that the effectiveness of such educational interventions depends not solely on technological infrastructure or curriculum design, but also on students’ psychological and motivational characteristics [5,6]. Understanding the cognitive and motivational mechanisms underlying students’ engagement with AI-based learning is therefore a prerequisite for the development and implementation of effective educational programmes in this domain [7,8].

Within theoretical frameworks of behaviour and technology adoption, researchers have emphasised predictive constructs that explain why individuals engage or fail to engage in technology-related learning activities. In this context, behavioural intention, as conceptualised in the Theory of Planned Behaviour (TPB) [9] and the Technology Acceptance Model (TAM) [7], is recognised as the most proximal determinant of actual behaviour. Accordingly, AI learning intention may serve as a critical indicator for predicting students’ active engagement in learning and applying AI technologies throughout their future professional practice.

Studies conducted among medical sciences students indicate that although general attitudes toward AI tend to be positive, concerns persist regarding ethical responsibility, algorithmic bias, data privacy, and the potential erosion of the human role in clinical decision-making [6,10]. This attitudinal ambivalence may directly or indirectly influence students’ willingness to learn about and adopt AI-related educational content [7,9]. Therefore, the accurate and evidence-based assessment of AI learning intention among medical sciences students is essential for informed curriculum design and data-driven educational policymaking.

In recent years, several psychometric instruments have been developed to assess different dimensions of engagement with AI. For instance, the Medical Artificial Intelligence Readiness Scale (MAIRS-MS) evaluates medical students’ readiness in terms of knowledge, skills, and attitudes toward AI(5) .The General Attitudes toward Artificial Intelligence Scale (GAAIS) measures positive and negative attitudes toward the societal and professional implications of AI [11]. Similarly, the Artificial Intelligence Attitude Scale (AIAS-4) focuses on general value-based attitudes toward AI [12], while the Artificial Intelligence Self-Efficacy Scale (AISES) assesses individuals’ perceived capability to learn and use AI applications [13].

Despite their contributions, these instruments primarily focus on readiness, attitudes, or self-efficacy rather than behavioural intention to learn AI. According to established behavioural frameworks, behavioural intention represents the most proximal predictor of actual engagement in educational activities. In this regard, the Artificial Intelligence Learning Intention Scale (AILIS) was specifically developed to directly operationalise and measure intention to learn AI. Grounded in motivational and technology acceptance models, AILIS assesses individuals’ willingness to engage in AI-related learning, pursue further training, and invest cognitive effort in this domain, and initial evidence has supported its construct validity and reliability [8].

However, the application of psychometric instruments across different cultural settings requires rigorous cross-cultural adaptation and validation because linguistic, educational, sociocultural, and professional differences may influence item interpretation, response styles, factor structure, and overall measurement performance [14,15]. Previous research has demonstrated that psychometric properties established in one population cannot be assumed to remain invariant across different cultural and educational contexts, particularly in rapidly evolving domains such as artificial intelligence education [16,17].

Although AI-related educational initiatives have increasingly emerged within Iranian medical and health sciences education programmes, no culturally adapted and psychometrically validated instrument is currently available to assess AI learning intention among medical sciences students 18,19. This limitation restricts the systematic evaluation of AI-related educational interventions, the identification of motivational determinants, and evidence-informed educational planning. Given the potential influence of cultural and educational factors on response patterns and factorial structure, rigorous validation of the Artificial Intelligence Learning Intention Scale (AILIS) within the Iranian context is warranted [14,15,20].

Therefore, given the growing importance of artificial intelligence in medical sciences education, the absence of a culturally validated instrument for assessing AI learning intention, and the need to ensure measurement validity within the Iranian context, the present study aimed to translate, cross-culturally adapt, and psychometrically evaluate the Artificial Intelligence Learning Intention Scale (AILIS) among medical sciences students.

## Methods

### Study design

This methodological study was conducted among students at Babol University of Medical Sciences, Iran. The study constituted part of a larger approved research project (Contract Code: 724136304) at the Health Research Institute of Babol University of Medical Sciences.

### Population and sampling

The study population comprised all undergraduate, master’s, and professional doctoral students enroled at Babol University of Medical Sciences. During the study period, a total of 2,227 students met the eligibility criteria, including 1,421 medical students, 462 dental students, and 344 nursing students. Given the heterogeneity of the population in terms of faculty affiliation and academic level, stratified random sampling with proportional allocation was employed to enhance representativeness and reduce sampling error. Initially, the target population was stratified based on two variables: faculty and academic level. The required sample size for each stratum was then determined proportionally to its share of the total student population to ensure that the final sample accurately reflected the underlying population structure. Following administrative coordination with the educational offices of the selected faculties, lists of eligible students within each stratum were obtained and used as sampling frames. Participants were subsequently selected through simple random sampling within each stratum, ensuring equal probability of selection for all eligible students.

The sample size was determined based on a commonly recommended guideline in psychometric research, which recommends recruiting at least 10 participants per item to ensure adequate statistical power for factor analysis and structural validation [21]. Because the original version of the Artificial Intelligence Learning Intention Scale (AILIS) consisted of 41 items, a minimum sample of 410 participants was required. To enhance the robustness of the psychometric analyses and compensate for potential data loss, a substantially larger sample of 800 medical sciences students was recruited. The sample was allocated proportionally across the predefined strata according to their relative representation within the total eligible population. It should be noted that sample size estimation was based on the original 41-item version of the scale prior to any item reduction procedures.

For construct validation, the total sample was randomly divided into two independent subsamples of equal size (*n* = 400 each). Exploratory factor analysis (EFA) was conducted using the first subsample to identify the underlying factor structure of the Persian AILIS, whereas confirmatory factor analysis (CFA) was subsequently performed using the second independent subsample to evaluate and confirm the factor structure identified in the EFA.

### Data collection process

Data collection was conducted in collaboration with the educational units of the participating faculties and scheduled to minimise disruption to students’ academic activities. From June 2025 to January 2026, eligible students were approached in person by the researcher following theoretical classes, practical training sessions, or clinical clerkships. After receiving a comprehensive explanation of the study aims and procedures, participants provided written informed consent. The study questionnaires were subsequently administered and completed during the same session, and all completed instruments were collected immediately upon completion.

### Inclusion and exclusion criteria

Inclusion criteria consisted of medical, dentistry, and nursing students who had completed at least one academic year, had prior experience using AI-based tools in theoretical and/or practical learning contexts, and expressed willingness to participate. These disciplines were selected due to their substantial engagement with AI applications in educational, clinical, and decision-support contexts and their central role in healthcare delivery and training. The exclusion criterion was incomplete completion of the study questionnaires.

### Instruments

Data were collected using the following instruments:

1. Demographic questionnaire.

The demographic form included variables such as age, gender, marital status, daily duration of AI use, academic level, year of university entry, field of study, level of internet access, and prior participation in AI-related workshops.

2. Artificial intelligence learning intention scale (AILIS).

The Artificial Intelligence Learning Intention Scale (AILIS), developed by Chai et al. (2024), consists of 41 items distributed across 11 factors organised into several conceptual dimensions. These dimensions encompass epistemic capacity (AI basic knowledge, programming efficacy, and designing AI for social good), facilitating environments (actual use of AI systems, subjective norms, and access to support and technology), psychological attitudes (resilience, optimism, and personal relevance), and psychological and behavioural outcomes (behavioural intention to learn AI and actual AI learning). The scale is scored using a five-point Likert format ranging from ‘strongly disagree’ to ‘strongly agree.’ In the original study, the internal consistency of the subscales ranged from Cronbach’s *α* = 0.80 to 0.90 [8]. cross-cultural adaptation of the AILIS were conducted in strict accordance with the guidelines proposed by Beaton et al. (2000) [14]. This standardised process included forward translation, synthesis of translations, back-translation, expert committee review, and pre-testing. The psychometric evaluation of the adapted Persian version was subsequently carried out following the methodological framework recommended by the COnsensus-based Standards for the selection of health Measurement INstruments (COSMIN) [22].

### Translation and cross-cultural adaptation of the persian version of the AILIS

Stage I: forward translation.

In the initial stage, the original English version of the AILIS was independently translated into Persian by two bilingual translators whose native language was Persian. One translator was familiar with the construct being measured, medical sciences education, and AI applications in learning contexts, and therefore focused on achieving conceptual and content equivalence. The second translator had no prior knowledge of the scale’s conceptual framework and no professional background in health or medical education. This naïve translation aimed to ensure linguistic naturalness, clarity, and comprehensibility for the target student population while identifying ambiguous or potentially misleading expressions in the original instrument. Each translator submitted their translated version along with a written report detailing challenging phrases, potential ambiguities, and the rationale behind lexical choices, thereby providing documented evidence to inform subsequent stages of the adaptation process.

Stage II: synthesis of the translations.

In this stage, the two forward translators and a recording observer convened to synthesise the translations. Using the original instrument alongside the two independent translations (T1 and T2), discrepancies, ambiguities, and wording differences were systematically reviewed and discussed. All decisions were reached through scientific consensus rather than individual preference. The entire synthesis process, including identified issues, deliberations, and final resolutions, was comprehensively documented in writing. This stage resulted in a single reconciled version of the instrument (T-12), which served as the basis for subsequent procedures.

Stage III: back-translation.

The synthesised Persian version (T-12) was independently back-translated into English by two translators who were blinded to the original instrument. Back-translation was conducted to verify translation accuracy and ensure that the adapted version accurately reflected the item content and conceptual meaning of the original scale.

The two back-translators (BT1 and BT2) were English language faculty members holding doctoral degrees in English linguistics. They had no prior knowledge of the constructs measured by the scale and were not informed about its conceptual background. Preferably, they also had no professional background in medical or health sciences. This approach minimised information bias and increased the likelihood of detecting subtle inconsistencies or unintended shifts in meaning within the synthesised translation.

Stage IV: expert committee review.

An interdisciplinary expert committee was established to achieve full cross-cultural equivalence. The committee consisted of the research team, two faculty members experienced in instrument development, and all translators involved in the forward and back-translation stages. The committee reviewed all available versions of the instrument, including the original scale, forward translations (T1 and T2), the synthesised version (T-12), back-translations (BT1 and BT2), and all written documentation describing prior decisions. Through comprehensive review and structured discussion, the committee reached consensus on all discrepancies and finalised a pre-final Persian version for field testing. During this stage, four types of equivalence were systematically evaluated:

Semantic equivalence: Ensuring that words and sentence structures conveyed identical meanings in both languages, with particular attention to grammatical accuracy and potential multiple interpretations.

Idiomatic equivalence: Identifying colloquial expressions or idioms that could not be directly translated and replacing them with culturally appropriate equivalents.

Experiential equivalence: Determining whether the situations or experiences described in the items were relevant and applicable within the target cultural context.

Conceptual equivalence: Ensuring that the underlying constructs carried the same conceptual meaning across cultures and were not altered by sociocultural differences.

If necessary, certain items underwent iterative forward and back-translation to optimise clarity and conceptual fidelity. All components of the instrument, including items and response options, were carefully reviewed. The committee also ensured that the pre-final version was comprehensible for university-level students in terms of readability and linguistic clarity.

Stage V: documentation and appraisal of the adaptation process.

In the final phase of cross-cultural adaptation, all documentation related to the translation and adaptation process was compiled for appraisal. This documentation included the original instrument, forward translations (T1 and T2), the synthesised version (T-12), back-translations (BT1 and BT2), the pre-final Persian version, and detailed written reports from each stage. The primary objective of this step was to audit the methodological rigour and transparency of the adaptation process, ensuring full adherence to recommended guidelines rather than modifying item content.

### Further testing of the adapted version

Although the primary objective of translation and cross-cultural adaptation is to preserve content equivalence and face validity between source and target versions, this process alone does not guarantee the preservation of the instrument’s psychometric properties within a new cultural context. According to COSMIN methodological standards, once an adapted version is finalised, its measurement properties must be systematically evaluated in accordance with its intended use.

### Psychometric evaluation of the persian version of the AILIS

Consistent with COSMIN recommendations, the psychometric properties of the Persian version of the AILIS were evaluated through a comprehensive, multi-stage process, including assessment of face validity, content validity, construct validity (exploratory and confirmatory factor analyses), convergent and discriminant validity, measurement invariance, internal consistency, and test–retest reliability.

### Face validity

Face validity was evaluated during the preliminary phase of the cross-cultural adaptation process using a small group of students from the target population. These participants were involved solely in face validity assessment and were not part of the main sample used for psychometric validation, which was conducted separately among 800 medical sciences students.

### Qualitative face validity

To assess qualitative face validity, the pre-final Persian version of the scale was administered to 10 students from the target population. Participants were asked to evaluate each item in terms of clarity of wording, simplicity of expression, comprehensibility of concepts, cultural appropriateness, and apparent relevance to the construct of AI learning intention. Written feedback was collected and systematically reviewed. Items identified as linguistically ambiguous, conceptually complex, or culturally incongruent were revised while preserving their original conceptual meaning and maintaining consistency with the source version.

### Quantitative face validity

Quantitative face validity was assessed among the same 10 students by evaluating the perceived importance of each item using a Likert scale. The Item Impact Score was calculated for each item to determine its perceived relevance. Items with lower impact scores were identified as having reduced perceived importance and were carefully reviewed for potential revisions in wording and conceptual clarity.

### Content validity

#### Qualitative content validity

Qualitative content validity was evaluated by a purposively selected panel of 10 experts whose areas of expertise were closely aligned with the dimensions of the construct being measured. The panel comprised three specialists in psychometrics and instrument development with extensive experience in the design, validation, and analysis of multidimensional measurement scales; two experts in medical education and AI-enhanced learning technologies with expertise in the educational and clinical applications of artificial intelligence; two specialists in medical ethics and health policy familiar with the ethical, legal, and societal implications of intelligent technologies; and three faculty members or clinical educators from various health sciences disciplines with practical experience in the use of AI-based systems for education and clinical decision-making.

The experts were asked to evaluate each item with respect to relevance, conceptual clarity, linguistic appropriateness, comprehensiveness, and cultural suitability for Iranian medical sciences students. Their written comments and recommendations were systematically reviewed, and appropriate modifications were made to improve item wording and cultural relevance while preserving the conceptual meaning and theoretical foundation of the original scale.

## Quantitative content validity

### Content validity ratio (CVR)

The necessity of each item was quantitatively evaluated using the Content Validity Ratio (CVR) according to Lawshe’s method [23]. The same panel of 10 experts rated each item on a three-point scale as ‘essential,’ ‘useful but not essential,’ or ‘not essential.’ CVR values were calculated using the formula CVR = (Ne − N/2)/(N/2), where *Ne* represents the number of experts who rated the item as essential and *N* represents the total number of experts. Based on Lawshe’s critical values, a minimum CVR of 0.62 was required for item retention when evaluated by 10 experts [23,24].

## Item-level content validity index (I-CVI)

Item relevance was assessed using the I-CVI. Experts rated each item on a four-point scale ranging from ‘not relevant’ to ‘highly relevant.’ The I-CVI was computed as the proportion of experts assigning a rating of 3 or 4 to each item. For panels consisting of six or more experts, an I-CVI value of ≥0.78 was considered acceptable [25].

## Scale-level content validity index (S-CVI/Ave and S-CVI/UA)

Content validity at the scale level was evaluated using two indices: S-CVI/Ave (the average of I-CVI values across all items) and S-CVI/UA (the proportion of items achieving universal agreement among experts). S-CVI/Ave is recommended as the preferred index in methodological studies due to its reduced sensitivity to panel size and greater stability in estimating overall content validity [26]. In contrast, S-CVI/UA represents a more stringent criterion and may underestimate scale-level validity in larger expert panels. Accordingly, Polit et al. recommend reporting S-CVI/Ave as the primary index and S-CVI/UA as a complementary descriptive measure [27]. An S-CVI/Ave ≥0.90 indicates excellent content validity at the scale level, while S-CVI/UA values ≥0.80 are generally considered acceptable [25,26]. Reporting both indices provides a more comprehensive assessment of expert agreement and content adequacy.

## Modified kappa statistic

To adjust for chance agreement among experts, the modified kappa coefficient (k*) was calculated for each item. First, the probability of chance agreement (Pc) was estimated based on the number of experts and the number assigning relevance ratings (scores of 3 or 4). The modified kappa was then computed. Interpretation followed the criteria proposed by Polit et al.: values ≤0.39 indicate poor agreement, 0.40–0.59 moderate agreement, 0.60–0.73 good agreement, and ≥0.74 excellent agreement beyond chance [27]. The use of modified kappa enhances methodological rigour by accounting for random agreement in expert judgement.

## Construct validity

### Exploratory factor analysis (EFA)

EFA was conducted to identify the underlying factor structure of the Persian AILIS. Prior to factor extraction, sampling adequacy was assessed using the Kaiser–Meyer–Olkin (KMO) index and Bartlett’s test of sphericity. KMO values ≥0.70 indicated adequate sampling, and a significant Bartlett’s test (*p* < 0.05) confirmed the suitability of the correlation matrix for factor analysis [28]. Factor extraction was performed using Principal Axis Factoring (PAF), which is recommended for psychometric data that may deviate from multivariate normality [29]. Given the theoretical expectation of correlated dimensions, an oblique Promax rotation was applied. The number of factors was determined based on eigenvalues greater than 1, scree plot inspection, and theoretical interpretability. Factor loadings ≥0.40 were considered acceptable [30].

### Exploratory graph analysis (EGA)

To strengthen evidence for construct validity and examine the dimensional structure more precisely, EGA was conducted as a complementary approach. Although not explicitly mandated by COSMIN, the framework supports data-driven methods for evaluating internal structure. EGA is a network psychometric method that identifies latent dimensions based on partial correlation patterns among items. In this study, a Gaussian Graphical Model was estimated using graphical LASSO regularisation, and resulting item clusters were interpreted as dimensions. Convergence between EGA-derived dimensions and those identified via EFA was considered additional empirical support for structural validity [31].

### Confirmatory factor analysis (CFA)

Confirmatory Factor Analysis was conducted to test the fit of the proposed measurement model. According to COSMIN, CFA provides the strongest evidence for structural validity [22]. Model fit was evaluated using multiple indices, including χ²/df, Comparative Fit Index (CFI), Tucker–Lewis Index (TLI), Root Mean Square Error of Approximation (RMSEA), and Standardised Root Mean Square Residual (SRMR) [32,33]. Values of CFI and TLI ≥ 0.90 and RMSEA and SRMR ≤ 0.08 were considered indicative of acceptable model fit [32].

### Convergent and discriminant validity

Convergent and discriminant validity were assessed within the CFA framework using variance-based indices. Convergent validity was evaluated by calculating Average Variance Extracted (AVE) and Composite Reliability (CR). AVE values ≥0.50 indicate that the construct explains at least half of the variance in its indicators, while CR values ≥0.70 reflect satisfactory internal consistency [28,34,35]. Discriminant validity was assessed using the Fornell–Larcker criterion, whereby the square root of AVE for each construct must exceed its correlations with other constructs [34].

### Measurement invariance by gender

Measurement invariance across gender was examined using Multi-Group Confirmatory Factor Analysis (MG-CFA) following standard structural equation modelling procedures [36]. Invariance was tested hierarchically across four levels: configural invariance (equal factor structure), metric invariance (equal factor loadings) and scalar invariance (equal item intercepts) [37]. Model comparisons were based on changes in fit indices rather than relying solely on chi-square difference tests due to their sensitivity to large sample sizes. Invariance was supported when changes in Comparative Fit Index (ΔCFI) did not exceed 0.01 and changes in RMSEA (ΔRMSEA) did not exceed 0.015 [38].

## Reliability

### Internal consistency

Internal consistency was assessed using Cronbach’s alpha (*α*) and McDonald’s omega (*ω*). Cronbach’s alpha values ≥0.70 were considered acceptable. Given the limitations of alpha under the assumption of tau-equivalence, McDonald’s omega was also calculated to provide a more robust reliability estimate, particularly for multidimensional instruments [39,40].

### Test–retest reliability

Test–retest reliability was evaluated using the Intraclass Correlation Coefficient (ICC) in a subsample of 30 participants who completed the instrument twice with a two-week interval. ICC values ≥0.70 were considered indicative of satisfactory temporal stability [41].

## Data analysis

EFA and preliminary statistical analyses were conducted using SPSS version 26. First- and second-order CFA were performed using AMOS version 24. Network analysis was conducted in R. Prior to conducting EFA and CFA, the data were screened for statistical assumptions. Univariate normality was assessed using skewness and kurtosis indices, while multivariate normality was evaluated using Mardia’s coefficient. Multivariate outliers were identified through Mahalanobis distance, and cases with associated probability values less than 0.001 were examined. According to Kline (2023), Mardia’s coefficient values exceeding 20 indicate violation of multivariate normality assumptions (34) .The extent and pattern of missing data were also examined. Given the limited proportion of missing values, Multiple Imputation was applied to handle missing data. Overall, assumption testing indicated that the dataset was suitable for factor analyses and subsequent statistical procedures.

## Results

### Participant characteristics

The findings indicated that the majority of participants (90.8%) were aged between 18 and 24 years. Regarding gender distribution, 54% were male. In terms of field of study, 52.62% were medical students. Most participants were single (97.3%). Concerning academic level, 65.37% were enroled in professional doctoral programmes. With respect to year of university entry, 30.8% were in their second academic year. Additionally, 87.9% reported no prior participation in AI-related training workshops (Table 1).

**Table 1.** Demographic characteristics of the study participants.

		Total = 800	
Variable	Category	n	%
Age (years)	18–24	726	90.8
	25–29	63	7.9
	≥30	11	1.4
Gender	Male	432	54.0
	Female	368	46.0
Field of Study	Medicine	421	52.62
	Dentistry	102	12.75
	Nursing	277	34.63
Marital Status	Single	778	97.3
	Married	22	2.7
	Divorced	0	0
Educational Level	Bachelor’s Degree	259	32.38
	Master’s Degree	18	2.25
	Doctor of Medicine (MD)/Professional Doctorate	523	65.37
Year of University Entry	Second Year	246	30.8
	Third Year	238	29.8
	Fourth Year	211	26.4
	Fifth Year and Above	105	13.1
Participation in AI Training Workshops	Yes	97	12.1
	No	703	87.9
Daily Internet Access (hours) Mean (SD)		(8.46)13.47	
Daily Use of AI for Academic Purposes (hours) Mean (SD)		(1.04)1.14	

### Face and content validity

The content validity of the Persian AILIS was evaluated using both qualitative expert assessment and quantitative indices, including the Item-Level Content Validity Index (I-CVI), the Scale-Level Content Validity Index (S-CVI/Ave), the Content Validity Ratio (CVR), and the modified kappa coefficient (k*). During the quantitative face validity assessment, two items *‘I can design computer programme to solve problems’* and *‘When an error occurs in a programme test, I can fix the programme’* obtained impact scores below the acceptable threshold of 1.5 and were therefore removed from the scale. In the quantitative content validity assessment, four additional items *‘I understand why AI technology needs big data for machine learning’*, *‘I can write computer codes without referring to examples’, ‘I can understand the basic logical structure of the programme’,* and *‘Using AI technology increases my productivity’* did not achieve the minimum acceptable CVR values based on Lawshe’s criteria. Consequently, these items were excluded from further analyses. Following the removal of these six items, the original 41-item scale was reduced to a 35-item version for subsequent psychometric evaluation. For the remaining items, all achieved an I-CVI ≥ 0.80, and the S-CVI/Ave was 0.96, indicating excellent scale-level content validity. In addition, the S-CVI/UA was 0.82, further supporting the adequacy of universal agreement among the expert panel. To control for chance agreement among experts, the modified kappa coefficient was calculated for each retained item, yielding values ranging from 0.61 to 0.88. According to the criteria proposed by Polit et al., these values reflect good to excellent agreement beyond chance, confirming strong expert consensus. Overall, the findings demonstrate that the refined 35-item Persian version of the AILIS is conceptually coherent, culturally appropriate, and methodologically robust for assessing AI learning intention in the target population.

### Exploratory factor analysis

Sampling adequacy for factor analysis was confirmed (KMO = 0.885), indicating that the data were appropriate for factor extraction. Bartlett’s test of sphericity was statistically significant (χ² = 7707.81, *p* < 0.001), demonstrating sufficient inter-item correlations to justify factor analysis.

**Figure 1.**
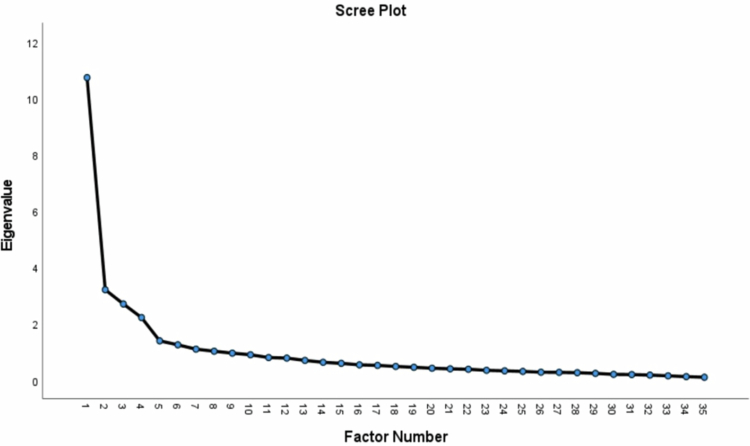
Scree plot for determining the number of latent factors in the exploratory factor analysis.

EFA results revealed that the first factor had an eigenvalue of 5.04 and accounted for 14.41% of the total variance, representing the largest contribution. The subsequent three factors explained 11.11%, 11.07%, and 7.96% of the variance, respectively. Collectively, the four factors accounted for 44.55% of the total variance of the AILIS. All items demonstrated acceptable factor loadings (>0.40) and loaded meaningfully on their respective factors. Within the Psychological Attitudes factor, the item ‘Using AI enhances my performance’ exhibited the highest factor loading (Table 2). The four-factor structure was further supported by inspection of the scree plot (Figure 1).

**Table 2.** Extracted factor structure of the AILIS based on exploratory factor analysis with Promax rotation.

Factor	Item	Factor Loading	Communalities	Variance %/Eigenvalue
Psychological Attitudes	18. Using AI helps me complete tasks faster.	0.820	0.610	14.41/5.04
	19. Using AI enhances my performance.	0.975	0.789	
	20. Using AI improves my effectiveness.	0.929	0.716	
	21. I feel hopeful about my future in a world where AI is widely used.	0.584	0.420	
	22. I focus on the positive aspects of issues in the emerging AI world.	0.531	0.322	
	23. In an AI-pervasive world, I expect the best even in uncertain times.	0.419	0.353	
	24. Overall, in an AI-driven world, I anticipate more positive than negative outcomes.	0.669	0.553	
	25. When I hear about AI advancements, I strengthen my intellectual capacity.	0.607	0.480	
	26. As I learn more about AI, I reflect on ways to develop myself.	0.653	0.467	
	27. In an AI-influenced world, I cultivate compassionate interpersonal relationships.	0.528	0.344	
	28. Facing an AI-emerging society, I focus on improving my service to others.	0.487	0.360	
Epistemic Capacity	1. I understand that AI uses machine learning to recognise patterns or objects.	0.584	0.345	11.11/3.89
	2. I understand that AI applications require training to produce accurate outputs.	0.970	0.856	
	3. I am aware that AI outputs must be carefully evaluated as they are probability-based.	0.816	0.649	
	4. I can conceptually envision how AI can support vulnerable populations.	0.731	0.601	
	5. I can generate ideas to improve life using AI.	0.836	0.690	
	6. I intend to contribute to designing AI that promotes human well-being.	0.516	0.371	
	7. I would participate in social initiatives to ensure AI is used for the public good.	0.664	0.514	
Facilitating Environments	8. My parents encourage me to engage in innovative AI learning activities.	0.827	0.664	11.07/3.87
	9. My instructors expect me to learn more about AI.	0.526	0.305	
	10. My classmates believe that learning AI is essential.	0.529	0.260	
	11. I can easily access information about AI.	0.647	0.431	
	12. I can continuously enhance my AI knowledge through open resources.	0.546	0.373	
	13. When I need to learn more about AI, I can easily find assistance.	0.621	0.429	
	14. I can download multiple AI applications to test them.	0.570	0.408	
	15. I have used AI-based translation systems.	0.656	0.471	
	16. I have interacted with AI chatbots such as Siri or Google Assistant.	0.478	0.342	
	17. I have used handwriting input features on smartphones or computers.	0.740	0.581	
Psychological and Behavioural Outcomes	29. I will continue learning AI in the future.	0.556	0.364	7.96/2.79
	30. I will pay attention to emerging AI applications.	0.447	0.224	
	31. I expect to remain concerned about AI development in the future.	0.464	0.327	
	32. I plan to allocate time to learning AI in the future.	0.429	0.362	
	33. I have intentionally searched for and watched AI educational videos.	0.827	0.658	
	34. I have reviewed a substantial amount of information about AI.	0.808	0.643	
	35. I have learned about AI through reading related materials.	0.735	0.533	

### Confirmatory factor analysis

**Figure 2.**
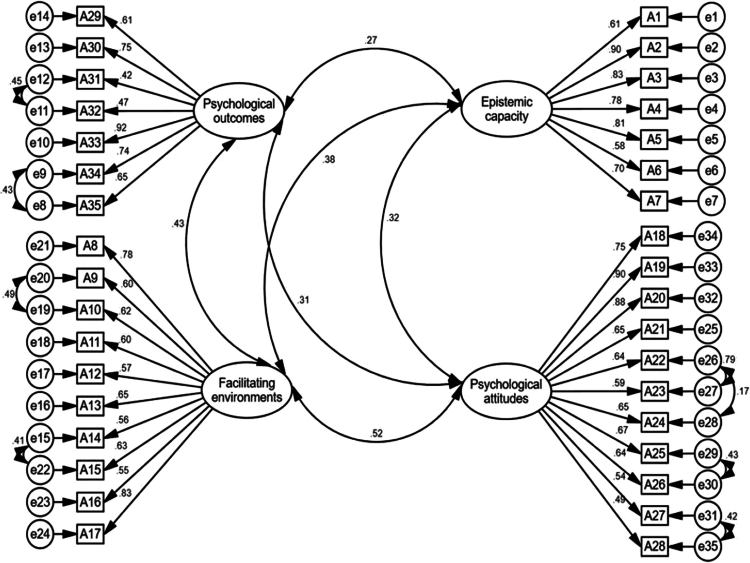
First-order confirmed factor structure of the AILIS.

The results of the first-order CFA indicated that the four-factor model of the AILIS demonstrated an acceptable fit to the data. The chi-square to degrees of freedom ratio (χ²/df) was 3.43, and the Root Mean Square Error of Approximation (RMSEA) was 0.078 (90% CI: 0.074–0.082). Additionally, incremental fit indices, including the Comparative Fit Index (CFI = 0.932), Incremental Fit Index (IFI = 0.933), and Goodness of Fit Index (GFI = 0.920), along with parsimonious fit indices, such as the Parsimony-Adjusted NFI (PNFI = 0.715) and Parsimony-Adjusted CFI (PCFI = 0.763), all indicated that the four-factor model exhibited an acceptable level of fit (Table 3). Examination of the inter-factor correlations in the first-order model revealed weak to moderate correlations, suggesting that the factors are sufficiently distinct and that the relative independence of the AILIS dimensions is maintained (Figure 2).

**Table 3.** Fit indices for the confirmatory factor analysis of the AILIS.

Fit Indices	χ^2^	Df	*P*-value	CMIN/df	RMSEA(CL90%)	PNFI	CFI	PCFI	IFI	GFI	SRMR	TLI
**First-order**	1874.87	546	<0.001	3.43	0.078 (0.074–0.082)	0.715	0.932	0.763	0.933	0.920	.079	.927
**Second-order**	1875.01	548	<0.001	3.42	0.078 (0.074–0.082)	0.718	0.932	0.766	0.933	0.921	.079	.927

**Figure 3.**
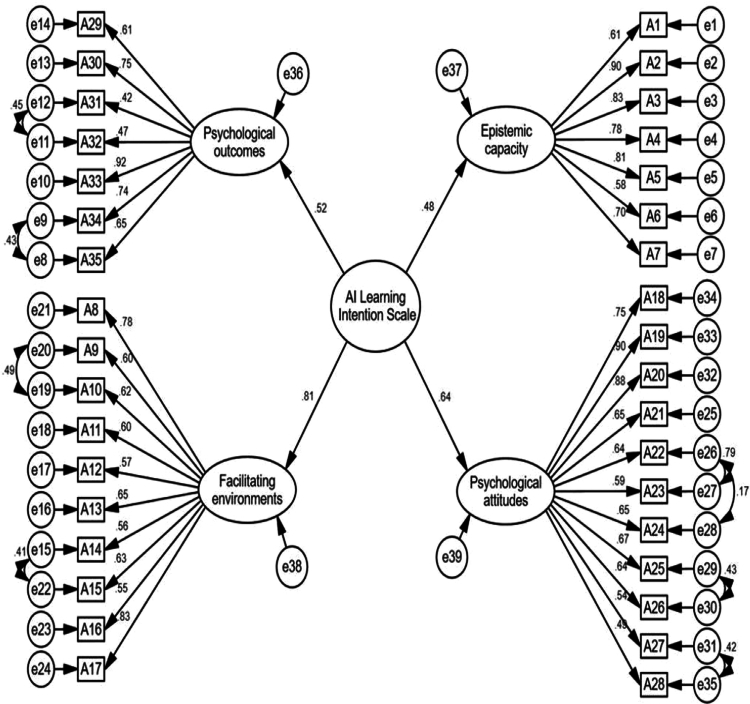
Second-order confirmed factor structure of the AILIS.

Subsequently, the results of the second-order CFA indicated that the proposed model also demonstrated a satisfactory fit to the data. The reported fit indices were: CMIN/df = 3.42, RMSEA = 0.078, CFI = 0.932, IFI = 0.933, GFI = 0.921, PNFI = 0.718, and PCFI = 0.766, all within acceptable ranges (Table 3). Each of the four first-order factors exhibited a significant association with the overall AILIS construct, contributing substantially to its explanation. These findings suggest that the shared variance among the four dimensions of AILIS is well accounted for by a single second-order construct. Accordingly, the four-factor structure of AILIS was confirmed within a second-order framework (Figure 3).

### Reliability, convergent, and discriminant validity

Cronbach’s alpha coefficients for the individual factors ranged from 0.854 to 0.904, while the overall scale demonstrated an alpha of 0.928, indicating good to excellent internal consistency for both the factors and the full instrument. McDonald’s omega values for the factors (0.854–0.899) and for the entire scale (0.923) further supported satisfactory internal reliability. The ICC values ranged from 0.750 to 0.860 for the factors and reached 0.821 for the overall scale, with all 95% confidence intervals falling within acceptable limits, indicating adequate temporal stability over the test–retest interval (Table 4).

Convergent validity was evaluated using CR and AVE. All factors demonstrated CR values above 0.84, reflecting strong internal consistency of the items within each construct. The AVE for the ‘Epistemic Capacity’ factor was 0.566, indicating good convergent validity. AVE values for the other factors Psychological Attitudes (0.468), Facilitating Environments (0.418), and Psychological and Behavioural Outcomes (0.451) were slightly below the recommended threshold of 0.50; however, given the high CR values across all factors, their convergent validity is considered acceptable. The overall pattern of indices indicates that the AILIS constructs possess appropriate conceptual distinctiveness. Differences between AVE and CR values among the factors, along with the confirmed four-factor CFA structure, suggest that each factor measures a distinct aspect of AI learning intention, collectively supporting satisfactory discriminant validity (Table 4).

**Table 4.** Convergent and Discriminant Validity, Internal Consistency, and Test–Retest Reliability of the AILIS.

Factor	*α*	*Ω*	ICC (95% CI)	CR	AVE
Psychological Attitudes	0.904	0.899	0.860 (0.770–0.894)	0.904	0.468
Facilitating Environments	0.874	0.870	0.798 (0.700–0.864)	0.876	0.418
Epistemic Capacity	0.893	0.893	0.790 (0.752–0.869)	0.899	0.566
Psychological and Behavioural Outcomes	0.854	0.854	0.750 (0.646–0.859)	0.845	0.451
Total	0.928	0.923	0.821 (0.751–0.872)	–	–

### Measurement invariance

The results demonstrated measurement invariance for the four-factor AILIS model across gender subgroups. Absolute changes in CFI and RMSEA for metric and scalar models, compared with the baseline model, were below 0.01 (Table 5), indicating that the scale functions equivalently for male and female participants.

**Tables 5.** Results of Multi-Group Confirmatory Factor Analyses across Different Subgroups.

Model	CFI	RMSEA	90% CI Lower	90% CI Upper	ΔCFI	ΔRMSEA
**Configural Invariance**	0.933	0.069	0.065	0.073	–	–
**Metric Invariance**	0.935	0.066	0.061	0.070	0.002	0.003
**Scalar Invariance**	0.926	0.072	0.067	0.075	0.007	0.003

### Exploratory network analysis

**Figure 4.**
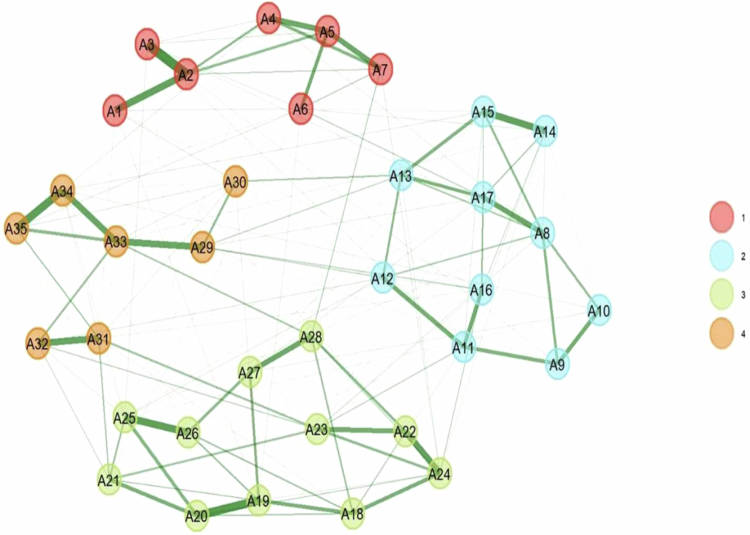
Factor structure of the AI Learning Intention construct based on network analysis.

The network analysis estimated the four-factor structure of the AILIS, confirming that all item dimensions corresponded with the factors identified in the EFA. In addition, a bootstrap analysis was conducted, generating 1,000 bootstrap samples. The results indicated that the median number of factors across the bootstrap samples was four, with a standard error of approximately 0.044 and a 92% confidence interval ranging from 3.14 to 7.01 factors. Importantly, all bootstrap samples retained four factors. Frequency analysis revealed that a four-factor structure was observed in 99.4% of the 1,000 bootstrap samples, with this configuration occurring 994 times. These findings suggest that the four-factor solution is highly stable and consistently reproduced across bootstrap samples (Figure 4).

### Scoring

The final Persian version of the AILIS consists of 35 items organised into four dimensions: epistemic capacity, facilitating environments, psychological attitudes, and psychological and behavioural outcomes. All items are rated on a five-point Likert scale ranging from ‘strongly disagree’ to ‘strongly agree,’ with each item scored from 1 to 5. The total score is calculated by summing the responses to all items, yielding a possible score range of 35 to 175. Higher total scores indicate a greater intention to learn artificial intelligence (AI).

Based on the total score distribution, AI learning intention can be interpreted across three levels. Scores ranging from 35 to 81 reflect a low level of AI learning intention, indicating limited motivation, weaker attitudes, or low perceived usefulness of AI learning. Scores between 82 and 128 represent a moderate level of intention, suggesting relatively positive attitudes and a moderate degree of readiness to engage in AI learning. Scores from 129 to 175 indicate a high level of AI learning intention, characterised by strong motivation, positive attitudes, high perceived usefulness, and an active willingness to learn and apply AI in future professional practice. In addition to the total score, each of the four dimensions can be calculated and interpreted separately, with higher scores within each domain reflecting a more favourable status in that specific aspect of AI learning intention.

## Discussion

This study aimed to conduct the cross-cultural adaptation and psychometric evaluation of the AILIS among medical sciences students. The EFA results indicated that the Persian version of the instrument demonstrated a coherent and interpretable four-factor structure within the context of medical education in Iran. Although Chai et al. (2024) reported 11 first-order factors, these factors are embedded within a broader hierarchical structure consisting of four higher-order dimensions [8]. From this perspective, the four-factor solution identified in the Persian version does not represent a deviation from the original theoretical model, but rather reflects a higher level of conceptual aggregation. In this study sampling adequacy and the significance of Bartlett’s test of sphericity confirmed that the item correlation matrix met the necessary conditions for extracting underlying factors, consistent with classical recommendations for factor analysis [29].

The extracted four-factor structure of the AILIS aligns with the multidimensional nature of the AI Learning Intention construct. This finding is consistent with Chai et al. (2024), who reported a four-factor structure with factor loadings exceeding 0.50 [8]. Although the proportion of variance explained in the Iranian sample was slightly lower than in the original study, this discrepancy may reflect cultural and educational differences among medical students [28,42]. The explained variance indicates that AI learning intention is not a simple, unidimensional construct; rather, it emerges from the complex interaction of attitudinal, cognitive, perceived utility, and behavioural dimensions, in line with the theoretical frameworks of TAM and TPB [7,9].

Among the extracted factors, the first factor, labelled ‘Psychological Attitudes,’ accounted for the largest proportion of explained variance and can be interpreted as the core of the AILIS construct. The prominence of this factor suggests that, within medical education, internal evaluations and cognitive-affective attitudes toward AI play a decisive role in shaping learning intention. This finding aligns with theoretical assumptions of TAM and TPB, which posit that a positive attitude toward the anticipated outcomes of a behaviour is the strongest predictor of behavioural intention [7,9]. Furthermore, the Unified Theory of Acceptance and Use of Technology (UTAUT) also emphasises that performance expectations and cognitive evaluations of technology’s usefulness significantly contribute to usage intention [43].

Within this factor, the item ‘Using AI enhances my performance.’ exhibited the highest factor loading. The theoretical significance of this item can be linked to the concept of perceived usefulness, identified in TAM as the most robust predictor of behavioural intention.(7) Perceived performance enhancement represents a high-level cognitive evaluation directly related to the professional logic of medical education, where efficiency, diagnostic accuracy, evidence-based decision-making, and improved patient outcomes are core values [1,43]. Therefore, for medical students, AI is meaningful and educationally legitimate only when it is perceived as a tool to enhance future professional performance, rather than merely an emerging technology or ancillary skill. This interpretation suggests that AI learning intention in this population is driven more by functional and professional evaluations than by technological curiosity. Another key finding was that all items demonstrated factor loadings above 0.40, and no items were removed. Methodologically, loadings in this range indicate adequate correlation of each item with its underlying factor and sufficient distinction from other factors [28,44]. These findings suggest that the translation and adaptation process was generally successful in preserving the conceptual structure of the instrument, although minor psychometric and structural differences were observed, which are expected in cross-cultural validation studies [14,15].

CFA results for both first- and second-order models demonstrated that the four-factor AILIS model exhibited acceptable fit with the Iranian data, with χ²/df, RMSEA, CFI, and IFI values all within recommended ranges, avoiding unnecessary model complexity [33,45]. Weak to moderate correlations among first-order factors indicated that the dimensions of AI learning intention are distinct yet interrelated, confirming the multidimensionality of the construct [28,32].The second-order model also showed good fit, indicating that the four first-order factors organise under a higher-order construct, reflecting a hierarchical structure that is convergent yet distinct from the overall AILIS construct [37,46].

The confirmation of the second-order structure is theoretically consistent with prevailing planned behaviour and technology acceptance models, where behavioural intention is considered an integrated construct emerging from the interaction of cognitive evaluations, attitudes, and perceived performance [9,43]. The convergence of the four AILIS dimensions under a single higher-order construct suggests that, in medical education, the intention to learn AI reflects the structured integration of multiple subjective evaluations regarding usefulness, psychological attitudes, and learning motivation. This pattern aligns with recent studies on digital technology acceptance in higher education, which show that intention to use or learn emerging technologies is multidimensional yet hierarchically organised [47,48]. These findings are also consistent with CFA and SEM results reported by Chai et al., where the four-factor CFA model confirmed the structure of AILIS, with fit indices such as CFI, TLI, and RMSEA in acceptable ranges [8].

Regarding reliability, the Persian AILIS demonstrated strong internal consistency and temporal stability [49–51], with items in each factor showing good convergence with their respective constructs. Even when AVE values were slightly below the recommended threshold, the consistently high CR values suggested an acceptable level of convergent validity. This pattern may indicate that, although the constructs are reliably measured, some items exhibit relatively lower shared variance with their intended latent factors, potentially reflecting a degree of item heterogeneity within certain dimensions. Such findings suggest that future refinements of specific items may further enhance the convergent validity of the scale. Overall, these results remain broadly consistent with those reported by Chai et al., supporting the reliability of the AILIS for assessing AI learning intention in the Iranian context [8,28,52].

Measurement invariance analysis indicated that the four-factor structure of the AILIS was stable across gender subgroups. Minimal changes in CFI and RMSEA for metric and scalar levels relative to the reference model indicate that the factors are interpreted equivalently by males and females, and the AI learning intention construct exhibits comparable conceptual and psychometric integrity [36,37]. This finding ensures that comparisons between groups in terms of means or inter-construct relationships are valid and free from gender bias, which is critical for educational research and evidence-based policy-making.

Finally, the bootstrapped network analysis, generating 1,000 samples, robustly confirmed the stability of the four-factor structure, with over 99% of samples replicating the four dimensions. This approach not only strengthens the validity of the factor structure but also demonstrates the instrument’s resilience and robustness in the face of random sampling and data variation [53,54]. The results further indicate that each AILIS dimension functions independently and distinctly, while converging with the overall AI learning intention construct, highlighting the added value of network analysis in evaluating structural stability.

The reliability evidence supports the internal consistency and stability of the Persian AILIS. The convergence of internal consistency indices indicates adequate item homogeneity while preserving the multidimensional nature of the construct. Consistent with contemporary psychometric recommendations, Cronbach’s alpha was complemented by McDonald’s omega to address the limitations of tau-equivalence assumptions and provide a more robust estimate of composite reliability [40,55]. In addition, test–retest reliability demonstrated satisfactory temporal stability, supporting the suitability of the instrument for both cross-sectional and longitudinal applications [49,56]. Compared with the original validation of the AILIS [8], which primarily relied on Cronbach’s alpha, the present study adopted a more comprehensive reliability framework by incorporating omega and test–retest reliability, thereby enhancing the methodological rigour of the psychometric evaluation.

## Conclusion

This study demonstrated that the Persian version of the AILIS exhibits a coherent and interpretable four-factor structure within the context of medical education in Iran, along with strong internal consistency, temporal stability, and adequate convergent and discriminant validity. Results from both first- and second-order CFA confirmed that, although the scale’s dimensions are interrelated, each dimension uniquely contributes to the overall AI learning intention construct and is well-integrated under a hierarchical higher-order structure. The translation and cross-cultural adaptation process was successful, with no items exhibiting semantic ambiguity or cultural inconsistency, thereby reinforcing the instrument’s validity in the Iranian context. Measurement invariance analyses indicated that the scale is stable across gender subgroups, while bootstrapped network analysis highlighted the robustness of the four-factor structure and the coherence of its dimensions.

### Strengths and limitations

This study employed a rigorous methodological design, including a large and diverse sample of medical, dental, and nursing students, and the use of multiple approaches to assess the validity and reliability of the scale, providing a robust psychometric framework for measuring AI learning intention. Key strengths include the comprehensive assessment of content and face validity through both qualitative and quantitative approaches, the calculation of the modified kappa coefficient to account for chance agreement among experts, and the evaluation of internal consistency using Cronbach’s alpha and McDonald’s omega. Temporal stability was also assessed via ICC, ensuring the reliability of the instrument over time. The concurrent use of diverse analytical methods, including exploratory and confirmatory factor analyses, convergent and discriminant validity evaluation, and multi-group invariance testing, further enhanced confidence in the instrument and supports the generalisability of the findings to similar educational contexts. Additionally, exploratory network and bootstrap analyses reinforced the stability of the four-factor structure and provided further evidence for the instrument’s structural validity.

Several limitations of the present study should be acknowledged. First, the sample was recruited from a single medical university, which may limit the generalisability of the findings to other universities, educational systems, and cultural contexts. Given the potential influence of institutional characteristics and variations in exposure to artificial intelligence education, caution is warranted when extending these findings to broader populations of medical sciences students.

Second, the use of self-reported data may introduce response-related biases, including social desirability and acquiescence bias. Although data were collected anonymously to reduce such effects, the possibility that participants provided socially desirable responses cannot be fully excluded.

Third, the present study did not assess external criterion-related validity or predictive validity. Therefore, the extent to which the Persian AILIS can predict actual engagement in AI-related learning behaviours or educational performance remains unclear. Future research should examine the predictive utility of the scale in relation to behavioural outcomes and real-world AI learning activities.

Finally, the cross-sectional design of the study limits the ability to examine changes in AI learning intention over time. Longitudinal studies are needed to better understand the stability and developmental trajectory of this construct among medical sciences students.

### Practical implications

The Persian version of the AILIS represents a valid and reliable instrument for assessing AI learning intention among medical students, with important implications for educational planning, curriculum development, and instructional improvement. At the institutional level, academic administrators and educational policymakers in medical schools can utilise this scale to identify students’ learning needs and to inform evidence-based development of AI-integrated curricula and educational strategies.

At the instructional level, educators in theoretical courses may use the findings to tailor teaching approaches according to students’ cognitive and attitudinal profiles, while clinical instructors and supervisors can design targeted workshops and practice-based learning activities that align with learners’ motivation and readiness to engage with artificial intelligence in clinical contexts.

Importantly, beyond the local context, the AILIS may also be applied in cross-national comparative studies and international research in digital and AI-enhanced medical education. Such applications can contribute to a better understanding of AI learning intention across diverse educational systems and support global efforts to strengthen the integration of intelligent technologies in health professions education.

## Data Availability

The dataset generated and analysed during the current study is not publicly available due to ethical restrictions and the presence of potentially identifying and sensitive participant information. Data are available from the corresponding author upon reasonable request and with approval from the Ethics Committee of Babol University of Medical Sciences.
